# AP2X-8 Is Important for Tachyzoite Growth and Bradyzoite Differentiation of *Toxoplasma gondii*

**DOI:** 10.3390/ani15091349

**Published:** 2025-05-07

**Authors:** Li-Xiu Sun, Meng Wang, Hany M. Elsheikha, Shi-Chen Xie, Bao-Quan Fu, Xing-Quan Zhu, Guo-Hua Liu

**Affiliations:** 1 Research Center for Parasites & Vectors, College of Veterinary Medicine, Hunan Agricultural University, Changsha 410128, China; sunlixiu1026@163.com; 2 State Key Laboratory for Animal Disease Control and Prevention, Key Laboratory of Veterinary Parasitology of Gansu Province, Lanzhou Veterinary Research Institute, Chinese Academy of Agricultural Sciences, Lanzhou 730046, China; wangmeng02@caas.cn (M.W.); xieshichen221@163.com (S.-C.X.); fubaoquan@caas.cn (B.-Q.F.); 3 School of Veterinary Medicine and Science, Faculty of Medicine and Health Sciences, University of Nottingham, Sutton Bonington Campus, Loughborough LE12 5RD, UK; hany.elsheikha@nottingham.ac.uk; 4 Laboratory of Parasitic Diseases, College of Veterinary Medicine, Shanxi Agricultural University, Taigu 030801, China

**Keywords:** *Toxoplasma gondii*, toxoplasmosis, AP2 transcription factors, AP2X-8, tachyzoite growth, bradyzoite differentiation

## Abstract

*Toxoplasma gondii* is a protozoan parasite capable of establishing chronic infections, with potential reactivation in immunocompromised individuals. However, the molecular mechanisms governing tachyzoite-to-bradyzoite differentiation remain incompletely understood. Previous studies have identified AP2 transcription factors as key regulators of this developmental switch. In this study, we investigated the role of the AP2 factor AP2X-8. Immunofluorescence analysis revealed that AP2X-8 is constitutively expressed in the nucleus of both tachyzoite and bradyzoite stages. Using CRISPR-Cas9-mediated homologous recombination, we successfully generated an *ap2X-8* knockout strain. Phenotypic assays including plaque formation, invasion, replication, and egress, and bradyzoite differentiation assays, were then performed to assess the impact of *ap2X-8* deletion. Our analyses showed that the loss of *ap2X-8* significantly impaired plaque formation and intracellular replication, while invasion and egress were unaffected. Furthermore, *ap2X-8* knockout enhanced bradyzoite differentiation in vitro. Despite these changes, deletion of *ap2X-8* did not alter parasite virulence in a mouse infection model. These findings demonstrate that AP2X-8 is an important regulator of *T. gondii* tachyzoite growth and bradyzoite differentiation, offering new insights into the parasite’s developmental regulation.

## Simple Summary

*Toxoplasma gondii* has a complex life cycle involving three main stages: oocysts (containing sporozoites), tachyzoites, and tissue cysts (containing bradyzoites). Understanding the mechanisms that regulate the transition between tachyzoites and bradyzoites has been a major focus of *T. gondii* research. Previous studies have identified several AP2 transcription factors, such as AP2IV-3, AP2IX-9 and AP2IX-4, as important regulators of this process. In this study, we investigated the role of another AP2 factor, AP2X-8. Using C-terminal endogenous tagging combined with immunofluorescence analysis, we determined that AP2X-8 is a nuclear protein constitutively expressed in both tachyzoite and bradyzoite stages. We then constructed an *ap2X-8* knockout strain using CRISPR-Cas9-mediated homologous recombination. Through a series of in vitro phenotypic assays, including plaque formation, invasion, replication, egress, and bradyzoite differentiation, we assessed the impact of *ap2X-8* deletion. Our results demonstrated that AP2X-8 promotes tachyzoite growth and suppresses the bradyzoite differentiation in vitro. These findings provide new insights into the regulatory role of AP2X-8 in *T. gondii* development and lay the groundwork for future investigations into parasite differentiation mechanisms.

## 1. Introduction

*Toxoplasma gondii* (*T. gondii*) is an obligate intracellular apicomplexan parasite that infects nearly all warm-blooded vertebrates, including humans. Chronic infection with *T. gondii* affects approximately one-third of the global population [1]. The parasite undergoes three major developmental stages: the rapidly replicating tachyzoite stage, the dormant tissue cyst stage containing bradyzoites, and the environmentally resistant oocyst stage containing sporozoites [2]. Oocysts are produced through sexual reproduction in the definitive feline host, where they develop within the intestinal epithelium and are shed into the environment via feces. Under warm, moist, and oxygen-rich conditions, oocysts sporulate and become infectious forms [3].

The asexual life cycle involves tachyzoites and bradyzoites. Tachyzoites disseminate systemically, eliciting robust immune responses. To evade immune clearance and drug treatment, some tachyzoites differentiate into bradyzoites, which persist in long-lived cysts primarily within brain and muscle tissues (skeletal and cardiac) [2]. The transition to slower-growing forms in spontaneous models occurs relatively early, approximately after ~20 tachyzoite divisions [4,5]. Early-stage parasites during this transition are predominantly slowly replicating tachyzoites, often referred to as pre-bradyzoites [6].

In immunocompetent individuals, *T. gondii* infection is usually asymptomatic or presents with mild, non-specific flu-like symptoms [7]. However, primary infection during pregnancy can lead to congenital toxoplasmosis, resulting in miscarriage, stillbirth, or congenital defects, such as mental retardation, blindness, and hydrocephalus. Immunocompromised individuals are particularly vulnerable to severe or reactivated infections [8]. The ingestion of tissue cysts in undercooked meat remains a major route of human infection [9].

Despite decades of research, the molecular mechanisms controlling bradyzoite development are not fully understood, and effective therapies targeting tissue cysts remain elusive [10]. Transcriptomic studies have revealed significant gene expression changes across *T. gondii* developmental stages and during the lytic cycle [11], implicating transcription regulation as a key driver of stage conversion. Apicomplexan parasites possess a unique family of transcription factors, the ApiAP2 proteins, which contain plant-like AP2 DNA-binding domains that regulate stage-specific gene expression [12].

Several AP2 factors have been functionally linked to the tachyzoite-to-bradyzoite transition [13]. For example, AP2IX-9 acts as repressor of bradyzoite differentiation; its overexpression inhibits tissue cyst formation, whereas its deletion enhances stage conversion [13]. AP2IX-9 binds to the CAGTGT motif and regulates critical bradyzoite genes, including *BAG1* and *B-NTPase* [13]. In contrast, AP2IV-3 acts as a transcriptional activator, enhancing tissue cyst formation upon overexpression [10]. Deletion of *ap2IV-4* results in the premature expression of bradyzoite proteins in tachyzoites and failure to form tissue cyst in the mouse brain [14]. Similarly, *ap2IX-4* deletion disrupts proper bradyzoite gene regulation, impairing cyst formation both in vitro and in murine models [15]. In this study, we investigated the role of AP2X-8, a factor previously identified through co-immunoprecipitation with the histone acetyltransferase GCN5b [16]. We found that AP2X-8 localizes to the nucleus in both tachyzoite and bradyzoite stages. Genetic ablation of *ap2X-8* significantly reduced parasite replication and overall growth. Moreover, the knockout of *ap2X-8* enhanced bradyzoite differentiation in vitro, but did not alter virulence in a mouse model. Our findings suggest that AP2X-8 acts as a negative regulator of bradyzoite differentiation in *T. gondii*.

## 2. Materials and Methods

### 2.1. Parasite Culture and Transfection

The tachyzoites of *T. gondii*, including PruΔ*ku80*Δ*hxgprt* (referred to as Pru), AP2X-8-6HA, and the *ap2X-8* knockout strains, were propagated in confluent monolayers of human foreskin fibroblast (HFF) cells (ATCC SCRC-104). HFF cells were maintained in Dulbecco’s modified Eagle medium (DMEM, Gibco, Suzhou, China) supplemented with 10% fetal bovine serum (FBS, Gibco, Auckland, New Zealand), 100 mg/mL streptomycin, and 100 U/mL penicillin, under 37 °C with 5% CO_2_ [17,18]. Approximately 5 × 10^5^ HFF cells were seeded per 25 cm^2^ culture flask (T25), and a confluent monolayer was established within 7 days. Tachyzoites were cultured in HFFs using DMEM supplemented with 1% FBS. When parasitophorous vacuoles (PVs) were densely packed with tachyzoites, parasites were mechanically released by passing through a 27-gauge needle and subsequently purified by filtration through a 5-µm polycarbonate membrane filter [18]. The purified tachyzoites were then used for infection, transfection, and sequencing experiments. Parasite transfection was performed using an ECM 830 Square Wave electroporator (BTX), following established protocols. Drug selection was initiated 36 h post transfection by adding 3 µM pyrimethamine, and clonal lines were isolated using the limiting dilution method [18].

### 2.2. Generation of Endogenously Tagged AP2X-8

Pru strain was genetically engineered to express AP2X-8 endogenously tagged at its C terminus with six tandem copies of the hemagglutinin (6×HA) epitope. To construct the CRISPR-Cas9 plasmid for endogenous tagging, a guide RNA targeting a region near the AP2X-8 stop codon was cloned into the pSAG1::Cas9-U6::sgUPRT plasmid, replacing the original guide RNA targeting the uracil phosphoribosyl transferase (*UPRT*) locus. The 6×HA-DHFR cassette was amplified as a single PCR product from the pLIC-6×HA-DHFR plasmid. The CRISPR plasmid and the amplified 6×HA-DHFR fragment were co-transfected into the Pru strain. Transfected parasites were selected with 3 µM pyrimethamine, and independent clones were isolated by limiting dilution [18]. Successful insertion of the 6×HA tag at the endogenous locus was confirmed by PCR and sequencing. The primers used for cloning and validation are presented in Supplementary Table S1.

### 2.3. Construction of ap2X-8 Knockout Strain

The *ap2X-8* gene was deleted from the Pru strain using a CRISPR-Cas9-based strategy. To generate *ap2X-8* knockout clones, a plasmid was constructed containing a DHFR selection cassette flanked by 1500 bp sequences homologous to the 5′ and 3′ regions of the *ap2X-8* locus. The 5′ and 3′ homologous arms were amplified from *T. gondii* genomic DNA, and the DHFR fragment was amplified from the pUPRT-DHFR-D plasmid. These three fragments were assembled into the pUC19 plasmid using the Clone Express II One Step Cloning Kit (Vazyme, Nanjing, China). A CRISPR plasmid targeting *ap2X-8* was constructed by replacing the UPRT-specific guide RNA in the pSAG1::Cas9-U6::sgUPRT with a guide RNA targeting *ap2X-8* locus [18]. The CRISPR plasmid and the homologous repair template (5′UTR-DHFR-3′UTR) were co-transfected into freshly egressed tachyzoites. Transfected parasites were selected with 3 µM pyrimethamine, and single PruΔ*ap2X-8* clone was isolated by limiting dilution. Successful deletion of the *ap2X-8* was confirmed by PCR. All primers used in the study are described in Supplementary Table S1.

### 2.4. Immunofluorescence Assay (IFA)

Parasite-infected HFFs were fixed with 4% paraformaldehyde (PFA) for 30 min, followed by washing in phosphate-buffered saline (PBS). Samples were permeabilized with 0.1% Triton X-100 in PBS for 20 min and then blocked with 3% bovine serum albumin in PBS for 2 h at room temperature. Primary antibodies were applied in blocking buffer and incubated either at 37 °C for 2 h or overnight at 4 °C, followed by washing with PBS. Secondary antibodies were then added and incubated for 1 h at 37 °C, with a final PBS wash. Nuclei were counterstained with DAPI (4′,6-diamidino-2-phenylindole, 1:1000 dilution, Invitrogen, Thermo Fisher Scientific, Waltham, MA, USA). Samples were mounted and visualized using a Leica confocal microscope (TCS SP8, Leica, Wetzlar, Germany). Image acquisition and analysis were performed using Leica Elements software (LAS_X_4.7.0) [18]. The primary antibodies used were rabbit anti-IMC1 (1:500 dilution, available in our laboratory) and mouse anti-HA (1:500 dilution, Invitrogen, Waltham, MA, USA). Secondary antibodies included goat anti-rabbit IgG (H + L) antibody conjugated with Alexa Fluor 488 (1:500 dilution, Invitrogen, Waltham, MA, USA), and goat anti-mouse IgG (H + L) antibody conjugated with Alexa Fluor 594 (1:500 dilution, Invitrogen, Waltham, MA, USA). For visualization of bradyzoite tissue cyst wall, donkey anti-rabbit IgG (H + L) antibody conjugated with Alexa Fluor 647 (1:500 dilution, Invitrogen, Waltham, MA, USA), goat anti-mouse IgG (H + L) antibody conjugated with Alexa Fluor 594 (1:500 dilution, Invitrogen, Waltham, MA, USA) and *Dolichos biflorus agglutinin* (DBA) (1:500 dilution, Vector Laboratories, Newark, CA, USA) were used.

### 2.5. Plaque Assay

Plaque assays were performed to evaluate parasite growth and propagation. Approximately 5 × 10^4^ HFF cells were seeded into 12-well tissue culture plastic plates and allowed to form confluent monolayers over 7 days. Tachyzoites from the Pru and PruΔ*ap2X-8* strains were then harvested from infected confluent HFFs in T25 flasks (Thermo Fisher Scientific, Waltham, MA, USA) and counted using a hemocytometer. A total of 200 freshly harvested tachyzoites were inoculated onto confluent HFF in 12-well plates (Thermo Fisher Scientific, Waltham, MA, USA) and incubated at 37 °C in a humidified atmosphere containing 5% CO_2_. After 9 days, the culture medium was removed, and the cells were fixed with 4% PFA for 30 min at room temperature. Fixed cells were stained with 0.5% crystal violet in PBS for 30 min then air-dried. Images of the plaques were captured using a camera, and plaque areas were quantified using Image J software (Image J 1.53k) [18]. The number of plaques was also recorded. Each experiment was performed independently three times, with three technical replicates per condition.

### 2.6. Invasion Assay

A two-color invasion assay was performed to assess the parasite invasion efficiency into HFFs. Approximately 5 × 10^4^ HFF cells were seeded into 12-well tissue culture plates and allowed to form confluent monolayers over approximately 7 days. Freshly harvested tachyzoites (~5 × 10⁶) from the Pru and PruΔ*ap2X-8* strains were inoculated into the HFF monolayers and incubated for 1 h at 37 °C. Following incubation, cells were fixed with 4% PFA for 30 min. Extracellular tachyzoites were first labeled by incubating samples with mouse anti-SAG1 antibody (1:500 dilution, available in our laboratory) at 37 °C for 1 h, followed by washing with PBS and incubation with goat anti-mouse IgG (H + L) antibody conjugated with Alexa Fluor 594 (1:500 dilution, Invitrogen, USA) for 1 h at 37 °C. After three washes with PBS, the cells were permeabilized with 0.1% Triton X-100 in PBS. A second round of staining was performed using rabbit anti-IMC1 antibody (1:500 dilution, available in our laboratory) for 1 h at 37 °C, followed by goat anti-rabbit IgG (H + L) antibody conjugated with Alexa Fluor 488 (1:500 dilution, Invitrogen, USA) for 1 h at 37 °C.

In this assay, extracellular tachyzoites were stained red, while both extracellular and intracellular tachyzoites were stained green [19,20]. Parasite invasion efficiency (%) was calculated using the formula:

$$\mathrm{Invasion}\;\mathrm{rate}\;\%=\frac{\left(\mathrm{green}\text{-}\mathrm{stained}\;\mathrm{tachyzoites})-(\mathrm{red}\text{-}\mathrm{stained}\;\mathrm{tachyzoites}\right)}{\;\mathrm{green}\text{-}\mathrm{stained}\;\mathrm{tachyzoites}\;}\times 100$$

Invasion efficiency was determined by counting the number of intracellular tachyzoites relative to the total number of tachyzoites across 20 random microscopic fields per sample for each strain [21]. Each experiment independently repeated three times, with three technical replicates per condition.

### 2.7. Intracellular Replication Assay

The intracellular replication assay was carried out to assess parasite proliferation efficiency following successful invasion. To examine the impact of *ap2X-8* deletion on *T. gondii* replication, approximately 5 × 10^4^ HFF cells were seeded into 12-well tissue culture plates and allowed to form confluent monolayers over approximately 7 days. Freshly harvested tachyzoites (~5 × 10^5^) from the Pru and PruΔ*ap2X-8* strains were then used to infect HFF monolayers for 2 h at 37 °C. After infection, uninvaded extracellular parasites were removed by washing with warm PBS, and the cells were cultured for an additional 34 h. Samples were then fixed with 4% PFA, permeabilized with 0.1% Triton X-100 in PBS for 20 min, and blocked with 3% BSA. For immunostaining, samples were incubated with rabbit anti-IMC1 (1:500 dilution, available in our laboratory) at 37 °C for 2 h, followed by three washes with PBS and incubation with goat anti-rabbit IgG (H + L) antibody conjugated with Alexa Fluor 488 (1:500 dilution, Invitrogen, USA) for 1 h at 37 °C. To assess replication, at least 150 PVs were analyzed per well to determine the number of intracellular tachyzoites per PV, as previously described [18]. Each experiment was independently repeated three times, with three technical replicates per condition.

### 2.8. Egress Assay

An induced egress assay was performed to assess the efficiency of parasite egress from HFFs and to examine the effect of *ap2X-8* deletion on *T. gondii* egress. Briefly, approximately 5 × 10^4^ HFF cells were seeded into 12-well tissue culture plastic plates and allowed to form confluent monolayers over approximately 7 days. Freshly harvested tachyzoites (~5 × 10^4^) from the Pru and PruΔ*ap2X-8* strains were used to infect the HFF monolayers and cultured for 48–60 h to achieve comparable number of parasites per PV. For egress induction, cells were then treated with 3 μM calcium ionophore A23187 in pre-warmed DMEM at 37 °C. After 3 min, egress was halted by immediately fixing the cells with 4% PFA, followed by permeabilization with 0.1% Triton X-100 in PBS. Intact PVs were stained with rabbit anti-GRA5 antibody (1:500 dilution, available in our laboratory), while parasites were labelled with mouse anti-IMC1 antibody (1:500 dilution, available in our laboratory). Subsequently, samples were incubated with goat anti-rabbit IgG (H + L) antibody conjugated with Alexa Fluor 488 and goat anti-mouse IgG (H + L) antibody conjugated with Alexa Fluor 594 (both at 1:500 dilution, Invitrogen, USA) for 1 h at 37 °C. At least 150 PVs were counted per well to determine the number of tachyzoites, as previously described [18]. Experiments were performed independently three times, each with three technical replicates.

### 2.9. Bradyzoite Differentiation Assay

Bradyzoite differentiation assays were performed as previously described [13,22]. Briefly, approximately 5 × 10^4^ HFF cells were seeded into 12-well tissue culture plates and allowed to form a confluent monolayer over seven days. Confluent HFFs were then infected with either Pru or PruΔ*ap2X-8* strains and cultured under standard conditions (37 °C, 5% CO_2_) for two days. After incubation, cells were fixed with 4% PFA, and permeabilized with 0.1% Triton X-100. Samples were subsequently incubated with rabbit anti-IMC1 (1:500 dilution, available in our laboratory) at 37 °C for 2 h, followed by three washes with PBS. The cells were then stained with FITC-conjugated DBA and goat anti-rabbit IgG (H + L) antibody conjugated with Alexa Fluor 594 (1:500 dilution, Invitrogen, USA) for 1 h at 37 °C. Bradyzoite differentiation was quantified by assessing the percentage of DBA-positive PVs from 150 randomly selected PVs per sample. Experiments were conducted independently three times, each with three technical replicates.

### 2.10. Mice and Virulence Assay

Six-to-eight-week-old female Kunming mice were obtained from the Center for Laboratory Animals of Lanzhou Veterinary Research Institute (LVRI) and acclimated for at least 1 week before experimentation. To evaluate the role of AP2X-8 in parasite virulence, mice were intraperitoneally injected with either 5 × 10^3^ or 5 × 10^5^ tachyzoites from the Pru and PruΔ*ap2X-8* strains. Each group (strain and infection dose) included six mice. Mice were monitored twice daily for up to 30 days to observe any symptoms of infection and determine the humane endpoints, as previously described [18].

## 3. Results

### 3.1. AP2X-8 Is Constitutively Expressed in the Parasite Nucleus

**Figure 1:**
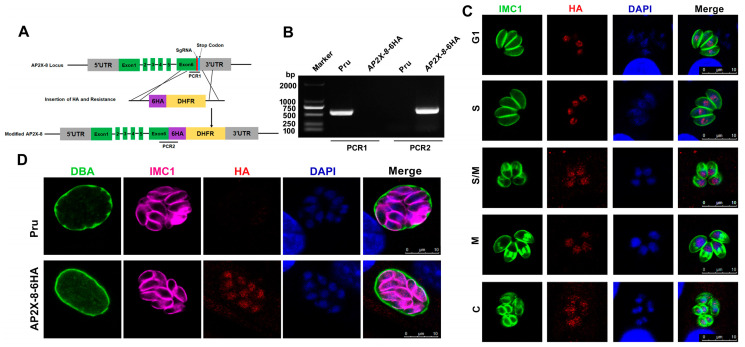
Construction and subcellular localization of AP2X-8 in *T. gondii*. (**A**) Schematic diagram illustrating the strategy for generating the AP2X-8 strain, with a 6×HA tag inserted at the C-terminus of the AP2X-8 gene in the Pru strain (indicated as AP2X-8-6HA). (**B**) Diagnostic PCRs confirming the successful insertion of the 6×HA. PCR1 detected the correct integration of the tag at the C-terminus of AP2X-8, while PCR2 confirmed the presence of the 6×HA sequence. (**C**) IFA showing constitutive expression of AP2X-8 throughout the cell cycle of tachyzoites. HFFs were infected with the AP2X-8-6HA strain for 36 h. IMC1 (green) marks different cell cycle stages—G1 (gap phase), S (synthesis phase), M (mitosis), and C (cytokinesis). AP2X-8-6HA was detected using an anti-HA antibody (red), and DNA was stained with DAPI (blue). (**D**) Subcellular localization of AP2X-8 in bradyzoites induced under alkaline stress for 2.5 days. The cyst wall was labelled with FITC-labelled DBA (green), parasites were stained with IMC1 (magenta), AP2X-8-6HA was detected with an anti-HA antibody (red), and DNA was visualized with DAPI (blue). Scale bars, 10 μm.

The full-length AP2X-8 gene (*TGME49_214960*) in *T. gondii* comprises six exons. Interestingly, no AP2 domain was detected in AP2X-8 based on predictions from the InterPro database (https://www.ebi.ac.uk/interpro/, accessed on 16 March 2024). To examine the expression pattern and subcellular localization of AP2X-8, we generated a strain with a C-terminal 6×HA tag inserted into the endogenous *ap2X-8* locus using CRISPR-Cas9 (Figure 1A). Correct integration of the tag was confirmed by PCR and DNA sequencing (Figure 1B). IFAs were performed to determine the localization and expression of AP2X-8 throughout the tachyzoite cell cycle, using the inner membrane complex-1 (IMC1) antibody to delineate cell cycle stages. The IFA results showed that AP2X-8 was constitutively expressed in the nucleus during all stages of the tachyzoite cell cycle, as indicated by colocalization of the HA signal with DAPI staining (Figure 1C). It is noteworthy that AP2X-8 was also constitutively expressed in the nucleus of bradyzoites after 2.5 days of alkaline medium induction (Figure 1D).

### 3.2. Construction of the ap2X-8 Knockout Strain by CRISPR-Cas9

**Figure 2:**
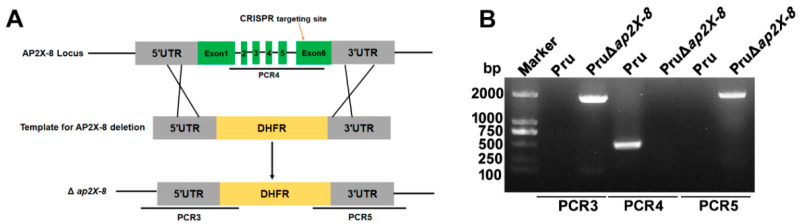
Construction of the *ap2X-8* knockout strain. (**A**) The PruΔ*ap2X-8* strain was generated using CRISPR-Cas9-mediated homologous recombination. (**B**) Diagnostic PCR analysis confirmed successful gene deletion. PCR3 and PCR5 were designed to detect the insertion of the homologous arms into the 5′ and 3′ regions of the *ap2X-8* locus, respectively. PCR4 was used to confirm the replacement of the *ap2X-8* coding region with the DHFR selection cassette.

To investigate the biological functions of AP2X-8, we disrupted the gene using a dihydrofolate reductase (DHFR) selection marker and a CRISPR-Cas9 mediated homologous recombination strategy. Successful generation of the PruΔ*ap2X-8* strain was confirmed by PCR analysis (Figure 2A). In accordance with the knockout strategy, the coding region of *ap2X-8* was undetectable in the PruΔ*ap2X-8* strain but was readily detected in the Pru strain (~500 bp) using diagnostic primer set PCR4 (Figure 2B). Further verification with PCR3 and PCR5 confirmed the amplification of ~1900 bp fragments in the PruΔ*ap2X-8* strain, which were absent in the Pru strain (Figure 2B), confirming successful replacement of the *ap2X-8* locus with the DHFR. These results demonstrate the successful generation of the *ap2X-8* knockout strain, enabling further phenotypic analysis.

### 3.3. AP2X-8 Is Essential for Tachyzoite Replication In Vitro

**Figure 3:**
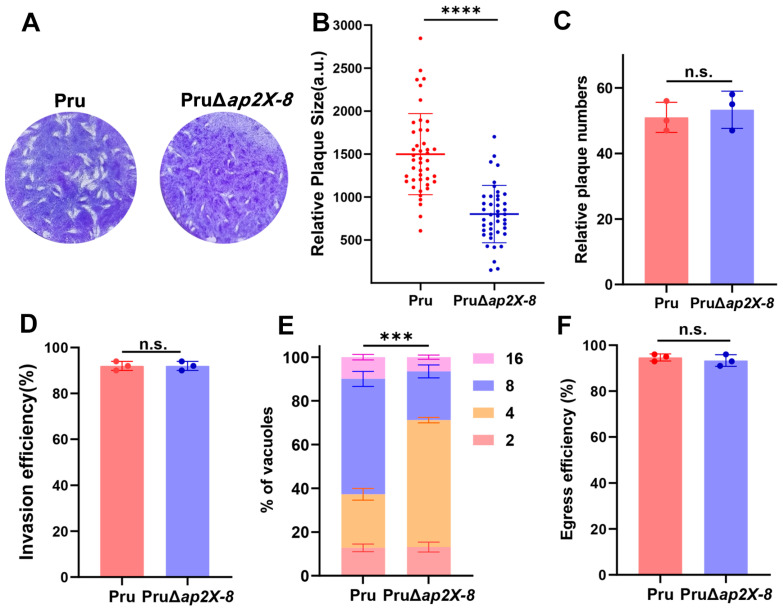
AP2X-8 is critical for *T. gondii* growth in vitro. (**A**) Representative images of plaques formed by Pru and PruΔ*ap2X-8* strains after 9 days of culture under standard conditions, showing smaller plaque sizes in the absence of *ap2X-8*. (**B**) Quantification of host cell monolayer lysis from plaque assays in (**A**), demonstrating that the absence of *ap2X-8* significantly inhibited tachyzoite growth (****, *p* < 0.0001). Each plaque is represented by a dot. (**C**) Quantification of plaque numbers formed by Pru and PruΔ*ap2X-8* strains, showing no significant difference between the two strains (n.s., *p* > 0.05). (**D**) Invasion efficiency assay showing no significant difference between Pru and PruΔ*ap2X-8* strains (n.s., *p* > 0.05). (**E**) Intracellular replication assay of Pru and PruΔ*ap2X-8* strains after 36 h of infection. The replication rate of the PruΔ*ap2X-8* strain was significantly lower than that of Pru strain (***, *p* < 0.001). At least 150 PVs per strain were analyzed. (**F**) Egress assay showing no significant difference between Pru and PruΔ*ap2X-8* strains (n.s., *p* > 0.05). Data represents the mean ± SD from three independent experiments. Statistical analysis was performed using a two-tailed, unpaired *t*-test (**B**–**D**,**F**) and a two-way ANOVA with Tukey’s multiple comparisons test (**E**).

To evaluate the role of AP2X-8 in parasite viability, we performed a standard plaque assay by comparing the Pru and PruΔ*ap2X-8* strains. After nine days of infection, the loss of *ap2X-8* resulted in significantly smaller plaque sizes relative to the Pru strain, while the number of plaques remained unchanged (Figure 3A–C). These results suggest that *ap2X-8* deletion impairs replication rather than invasion. To further investigate this phenotype, we conducted invasion, replication and egress assays. Tachyzoites were purified by syringe lysis and filtration and subjected to an attachment and invasion assay [23]. After 1 h of incubation, both Pru and PruΔ*ap2X-8* tachyzoites exhibited similar invasion rates, with approximately 90% of parasites successfully invading host cells (Figure 3D).

We then conducted a replication assay, which revealed that the replication rate of the PruΔ*ap2X-8* strain was significantly reduced compared to the Pru strain, as indicated by an increased number of vacuoles containing only four parasites and a reduced number of vacuoles containing eight parasites (Figure 3E).

Finally, we evaluated parasite egress using Ca^2+^ ionophore-induced calcium egress assay, which showed no significant difference between the Pru and PruΔ*ap2X-8* strains (Figure 3F). These results indicate that AP2X-8 is critical for sustaining *T. gondii* growth by promoting efficient intracellular replication in vitro.

### 3.4. Loss of ap2X-8 Induces Bradyzoite Differentiation

**Figure 4:**
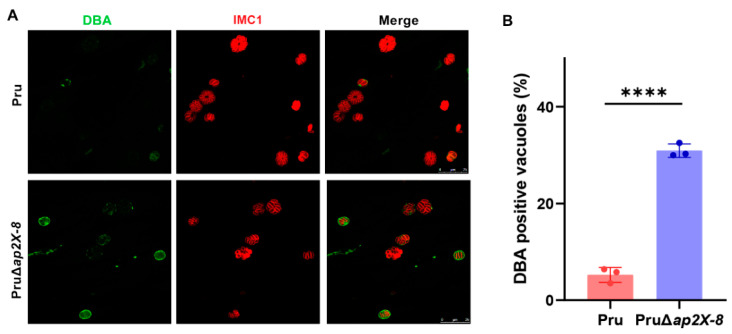
Knockout of *ap2X-8* promotes tachyzoite-to-bradyzoite conversion under standard culture conditions. (**A**) Representative immunofluorescence images of vacuoles formed by Pru and PruΔ*ap2X-8* strains after 2 days of culture under standard conditions. Parasite cyst walls were stained with DBA (green), and the parasites were stained with IMC1 (red). Scale bar, 25 μm. (**B**) Quantification of bradyzoite differentiation in Pru and PruΔ*ap2X-8* strains after 2 days under standard culture conditions. Deletion of *ap2X-8* significantly increased the spontaneous conversion of tachyzoites to bradyzoites (*p* < 0.0001). Data represents the mean ± SD from three biological replicates, with the percentage of DBA positive PVs calculated from at least 150 PVs per replicate. Statistical significance was determined using a two-tailed, unpaired *t*-test, ****, *p* < 0.0001.

Previous studies have demonstrated that AP2 factors are involved in the conversion of tachyzoites into bradyzoites [13]. To determine whether AP2X-8 contributes to this process in vitro, we performed a bradyzoite differentiation assay. HFFs were infected by Pru or PruΔ*ap2X-8* strains and cultured under standard conditions for 2 days. Differentiating parasites were stained with anti-HA (red) to label the parasites and DBA (green) to detect the tissue cyst wall. The results showed that deletion of *ap2X-8* significantly increased tissue cyst formation compared to the Pru strain under standard culture conditions (Figure 4A,B).

### 3.5. Deletion of ap2X-8 Does Not Affect the Virulence of T. gondii in Mice

**Figure 5:**
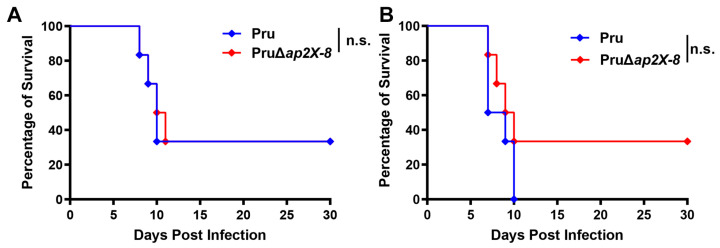
Virulence of Pru and PruΔ*ap2X-8* strains in vivo. (**A**,**B**) Survival curves of Kunming mice infected by either Pru or PruΔ*ap2X-8* strains. Groups of mice were intraperitoneally inoculated with 5 × 10^3^ tachyzoites (**A**) or 5 × 10^5^ tachyzoites (**B**), and survival was monitored for 30 days. No significant difference in survival was observed between the two groups. Survival data were analyzed using the Gehan–Breslow–Wilcoxon test. n.s., not significant, *p* > 0.05.

Previous studies have shown that the deletion of certain AP2 factors can influence the virulence of *T. gondii* in mice. To determine whether the loss of *ap2X-8* affects parasite virulence, we performed a mouse infection assay using either 5 × 10³ or 5 × 10^5^ tachyzoites from the Pru strain or the PruΔ*ap2X-8* mutant strain. Mice were monitored for survival over a 30-day period. The results revealed no significant differences in survival between mice infected with the Pru strain and those infected by PruΔ*ap2X-8* strain, regardless of the inoculum size (Figure 5A,B). These findings indicate that deletion of *ap2X-8* does not affect the virulence of *T. gondii* in mice.

## 4. Discussion

*Toxoplasma gondii* uundergoes major phenotypic changes throughout its life cycle. Recent studies have identified several AP2 transcription factors as critical regulators that orchestrate transcriptional switches necessary for the parasite’s morphological differentiation [10,13,14,15]. The establishment of tissue cysts within host cells is essential for *T. gondii* transmission and underlies chronic infection in humans. Therefore, understanding the role of AP2 factors in bradyzoite differentiation is crucial for the prevention and control of toxoplasmosis.

In the present study, we investigated the role of the AP2 factor AP2X-8, which was found to be localized to the nucleus and to play an important role in *T. gondii* growth. Deletion of *ap2X-8* enhanced bradyzoite formation in vitro but did not affect parasite virulence in mice. Similar to other characterized AP2 factors, such as AP2IV-4, AP2IV-3, and AP2IX-9, AP2X-8 is exclusively localized to the nucleus during both tachyzoite and bradyzoite stages [10,13,14,15]. Although many AP2 factors act independently or form homo- or heterodimers to perform biological functions [10], AP2X-8 is unusual in that it lacks a typical AP2 domain. This suggests that it may cooperate with other AP2 factors, such as AP2IX-7, AP2VIIa-5, or AP2XII-4 [10,13,14,15], all of which are known to interact with GCN5b—a chromatin-remodeling enzyme essential for *T. gondii* growth and development [24].

To further characterize AP2X-8, we generated *ap2X-8* knockout parasites in the type II *T. gondii* Pru strain and conducted phenotypic analyses assessing plaque formation, invasion, proliferation, and egress. Deletion of *ap2X-8* resulted in impaired tachyzoite replication, demonstrating that AP2X-8 promotes parasite growth in vitro. These findings are consistent with previous studies showing that other AP2 factors regulate *T. gondii* proliferation. For example, deletion of *ap2IX-5* abolished plaque formation and completely blocked tachyzoite proliferation [25,26], while loss of AP2XII-9 led to severe downregulation of IMC and apicoplast proteins, impairing daughter bud formation and cell division [27].

Interestingly, the deletion of *ap2X-8* also enhanced bradyzoite differentiation in vitro, suggesting that AP2X-8 functions as a negative regulator of the tachyzoite-to-bradyzoite transition. Several AP2 factors have been previously implicated in this conversion. For instance, AP2IX-9 acts as a repressor of bradyzoite gene expression by binding cis-regulatory elements such as the *BAG1* promoter [13], while AP2IV-3 functions as an activator, with *ap2IV-3* knockout resulting in reduced tissue cyst formation [10]. Interestingly, the expression of key bradyzoite genes, including *BAG1*, is regulated by multiple AP2 factors (AP2XI-4, AP2IV-3, AP2IX-9, and AP2IX-4) [10], highlighting a competitive regulatory network that likely enables *T. gondii* to finely tune its developmental response to environmental conditions.

Recent studies have also linked iron metabolism to stage conversion. Iron is essential for *T. gondii* survival, but excessive iron disrupts the parasite’s redox balance, while iron depletion promotes bradyzoite differentiation [28]. Additionally, the regulator BFD1 has been shown to drive bradyzoite development by binding transcriptional start sites of differentiation-associated genes; its expression alone is sufficient to induce differentiation even without external stress [29]. BFD2 has been identified as an upstream regulator required for full induction of BFD1, creating a positive feedback loop essential for chronic stage formation [30]. A recent study of our group revealed that the transcription factor AP2XI-2 not only plays an essential role in *T. gondii* merogony as a key negative regulator, but it is also an important virulence factor because deletion of *ap2XI-2* significantly attenuated the parasite virulence [18]. However, the present study demonstrated that the deletion of *ap2X-8* did not affect parasite virulence in mice, indicating that PruΔ*ap2X-8* is not a promising vaccine candidate. Nonetheless, our findings establish AP2X-8 as an important regulator of *T. gondii* growth and bradyzoite differentiation.

## 5. Conclusions

This study demonstrates that AP2X-8 is exclusively localized to the nucleus during both tachyzoite and bradyzoite stages. Functional assays revealed that AP2X-8 promotes tachyzoite replication while suppressing bradyzoite differentiation in vitro. Furthermore, virulence assays confirmed that *ap2X-8* deletion does not alter *T. gondii* virulence in mice. Together, our findings highlight a novel regulatory role for AP2X-8 in stage conversion and offer new insights into the molecular mechanisms underlying *T. gondii* development.

## Data Availability

The original contributions presented in this study are included in the article. Further inquiries can be directed to the corresponding authors.

## Supplementary Materials

The following supporting information can be downloaded at: https://www.mdpi.com/article/10.3390/ani15091349/s1. Table S1: Primers used in the study.
