# Supplementary material for: AP2X-8 Is Important for Tachyzoite Growth and Bradyzoite Differentiation of Toxoplasma gondii
Source: Animals (Basel). 2025 May 7;15(9):1349. doi: 10.3390/ani15091349 (PMC12070893; doi:10.3390/ani15091349)
Supplement: Supplementary file 1 [file animals-15-01349-s001.zip › animals-3577255-supplementary.pdf]

Supplementary Table S1: Primers used in the study.

Primer names	Primer sequence (5′-3′)	Use
SgRNA-AP2X-8-Tag	GAGAGGCGCGGGGGGATAGC	SgRNA of the CRISPR plasmid for tagging AP2X-8 with 6HA
SgRNA-AP2X-8-Tag-Fw	GAGAGGCGCGGGGGGATAGCGTTTTAGAGCTAGAAATAGC	Construction of the CRISPR plasmid for tagging AP2X-8 with 6HA
SgRNA-AP2X-8-Tag-Rv	AACTTGACATCCCCATTTAC	Construction of the CRISPR plasmid for tagging AP2X-8 with 6HA
AP2X-8-HR-Fw	CCCATTCAGCTGGACTGGAGTAAAGTGAGAGGCGCGGGGGGAGCTAGCAAGGGCTCGGG	Amplification for 6HA-DHFR fragment with the homologous arms of AP2X-8
AP2X-8-HR-Rv	CTCGTGCCACCTGTTTTCTCTGAAATCTTTCTTCGTCCGGCTATACGACTCACTATAGG	Amplification for 6HA-DHFR fragment with the homologous arms of AP2X-8
PCR1-AP2X-8-Tag-Fw	CGTTTCCCACTCTGACATCTT	Detection for the replacement of C-terminal AP2X-8 by 6HA fragment in PCR1
PCR2-AP2X-8-Tag-Fw	CGTTTCCCACTCTGACATCTT	Detection for the insert 6×HA fragment in PCR2
PCR1-AP2X-8-Tag-Rv	TCTCATTTCCCTTGTTTCCTT	Detection for the replacement of C-terminal AP2X-8 by 6HA fragment in PCR1
PCR2-AP2X-8-Tag-Rv	ATTATACCCGTGTGTTACG	Detection for the insert 6×HA fragment in PCR2
SgRNA-AP2X-8-KO	GCCTACATGCAACAAGACAC	SgRNA of the CRISPR plasmid for deleting AP2X-8
SgRNA-AP2X-8-KO-Fw	GCCTACATGCAACAAGACACGTTTTAGAGCTAGAAATAGC	Construction of the CRISPR plasmid for deleting AP2X-8
SgRNA-AP2X-8-KO-Rv	AACTTGACATCCCCATTTAC	Construction of the CRISPR plasmid for deleting AP2X-8
U5-AP2X-8-Fw	GGTTTTCCCAGTCACGACGTTATTTTATCTCGACATGACACGCTT	Amplification for the 5' homologous arms of AP2X-8 to construct the pUPRT-DHFR-D plasmid
U5-AP2X-8-Rv	GGATTTACAGCCTGGCGAAGCTTTCTCCTGTGCCAGGCAACCTC	Amplification for the 5' homologous arms of AP2X-8 to construct the pUPRT-DHFR-D plasmid
U3-AP2X-8-Fw	CTATGCACTTGCAGGATGAATTCCGTTTCCCACTCTGACATCTT	Amplification for the 3' homologous arms of AP2X-8 to construct the pUPRT-DHFR-D plasmid
U3-AP2X-8-Rv	GAGCGGATAACAATTTCACACGTCTTGTGACTGAACCGTAG	Amplification for the 3' homologous arms of AP2X-8 to construct the pUPRT-DHFR-D plasmid
DHFR-Fw	AAGCTTCGCCAGGCTGTAAATCC	Amplification for the DHFR fragment to construct the pUPRT-DHFR-D plasmid
DHFR-Rv	GAATTCATCCTGCAAGTGCATAG	Amplification for the DHFR fragment to construct the pUPRT-DHFR-D plasmid
pUC19-Fw	TGTGAAATTGTTATCCGCTC	Amplification for the pUC19 fragment to construct the pUPRT-DHFR-D plasmid
pUC19-Rv	AACGTCGTGACTGGGAAAACC	Amplification for the pUC19 fragment to construct the pUPRT-DHFR-D plasmid
PCR3-AP2X-8-Fw	CAACAGAGAAGTGTTGAGCACAGG	Detection for the insertion of 5' homologous fragment of AP2X-8 in PCR3
PCR3-AP2X-8-Rv	GCCAAAGTAGAAAGGAATTAGCAT	Detection for the insertion of 5' homologous fragment of AP2X-8 in PCR3
PCR4-AP2X-8-Fw	TTTTGGTGATTTGCGTTTGTA	Detection for the deletion of AP2X-8 in PCR4
PCR4-AP2X-8-Rv	AGTCATCCTTCTCGCCTTCTT	Detection for the deletion of AP2X-8 in PCR4
PCR5-AP2X-8-F	TGACGCAGATGTGCGTGTATCCAC	Detection for the insertion of 3' homologous fragment of AP2X-8 in PCR5
PCR5-AP2X-8-R	CCACAACACCACATCAACACCGTT	Detection for the insertion of 3' homologous fragment of AP2X-8 in PCR5
AP2X-8-KZ-Fw	ATTTTATCTCGACATGACACGCTT	Amplification for the 5UTR-DHFR-3UTR fragment of AP2X-8
AP2X-8-KZ-Rv	CGTCTTGTGACTGAACCGTAG	Amplification for the 5UTR-DHFR-3UTR fragment of AP2X-8
